# The conversion of native savannah into pasturelands does not affect exclusively species diversity: Effects on physiological condition of a highly abundant dung beetle species

**DOI:** 10.1002/ece3.10752

**Published:** 2023-11-20

**Authors:** César M. A. Correa, Kalel Caetano da Silva, Pedro Lucas Moreira de Oliveira, Renato Portela Salomão

**Affiliations:** ^1^ Laboratório de Bioecologia de Scarabaeoidea (Scaralab) Universidade Estadual de Mato Grosso do Sul Aquidauana Brazil; ^2^ Universidade Federal de Mato Grosso do Sul Aquidauana Brazil; ^3^ Facultad de Estudios Superiores Iztacala Universidad Nacional Autónoma de México Tlalnepantla Mexico; ^4^ Pós‐graduação em Ecologia Instituto Nacional de Pesquisas da Amazônia Manaus Brazil

**Keywords:** biodiversity conservation, bioindicators, Brazilian Cerrado, physiology, Scarabaeinae

## Abstract

Dung beetles are efficient indicators to obtain responses regarding the effects of land use change on biodiversity. Although the biological consequences of Cerrado conversion into pasture have been observed at the assemblage scale, there are no cues regarding the effects of tropical savanna conversion into pasture on physiological condition of dung beetle individuals. In this study, we evaluated whether native and non‐native habitats in Cerrado affect the physiological condition and body traits of males and females of *Phanaeus palaeno*. The individuals were collected from a Cerrado fragment (sensu stricto) and an exotic pasture (*Urochloa* spp.). Physiological condition was assessed through the estimation of individuals' dry body mass, fat mass, and muscle mass. Body traits were estimated through individual body size and males' horn length. We did not find differences between dung beetle morphological traits between Cerrado and pastures. However, individuals collected in exotic pastures had lower dry mass and fat mass, but higher muscle mass, than in conserved Cerrado. Understanding how the land use change affects individuals' body condition is essential to maintain abundant and healthy populations of dung beetles in human‐modified landscapes. Although the estimation of physiological condition is logistically more complex than species body traits, future studies aiming to present complex and finer ecological responses of dung beetles should incorporate physiological data to their approaches.

## Graphical abstract

Habitats determine individual physiological condition. Physiological responses to habitat type are sex dependent in a tropical dung beetle species.

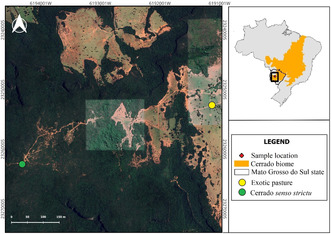

## 1 INTRODUCTION

The physiological condition of the organisms stands out as an efficient approximation of the effect of environmental quality on the individuals' condition (Capolupo et al., 2017; Cooke et al., 2013; Salomão et al., 2020). While environmental transformation may take years to present clear effects on biodiversity (Bennett & Saunders, 2010; Haddad et al., 2015), studies of physiological condition can present rapid responses (Cooke et al., 2013), which can anticipate future decreases in native animal populations (Fefferman & Romero, 2013; Wikelski & Cooke, 2006). Among the different attributes used to analyze physiological condition, body mass is a proxy of organism overall reflecting individual fitness (Briffa & Sneddon, 2007; Córdoba‐Aguilar et al., 2016). In addition, individual muscle mass is directly related to sexual coercion, while fat mass is a proxy of individuals' energetic reserves (Droney & Hock, 1998; Marden & Cobb, 2004; Schulte‐Hostedde et al., 2005). Thus, through the study of different physiological attributes, it is possible to understand the effects of environmental change on the individual's health and population dynamics.

To obtain rapid and reliable responses regarding the effects of land use change on biodiversity, terrestrial invertebrates have been used as bioindicators (Borges et al., 2021; Gerlach et al., 2013). Among them, dung beetles (Coleoptera: Scarabaeinae) have gained attention as efficient indicators of environmental changes (França, Louzada, et al., 2016; Halffter & Favila, 1993; Nichols et al., 2007). In addition, dung beetles play a vital role in upholding ecosystem functioning. Through its behavior of burying portions of food (decaying organic matter) into the soil for nesting and feeding their offspring (Halffter & Edmonds, 1982), dung beetles contribute to bioturbation, nutrient cycling, control of parasites, and secondary seed dispersal (Nichols et al., 2008). Most ecological studies assess the effect of environmental quality and anthropogenic landscapes on dung beetle assemblages, presenting a trend of decreases of diversity in less conserved sites (Halffter & Favila, 1993; López‐Bedoya et al., 2022; Nichols et al., 2007). However, few studies have analyzed dung beetle physiology, demonstrating a general negative response in individuals' physiological conditions when subjected to environmental disturbances in tropical forests, such as selective logging (França, Barlow, et al., 2016), fragmentation (Salomão et al., 2018), and urbanization (Salomão et al., 2020). To the date, these studies are exclusively focused on tropical rainforests, and no study evaluated how the land use changes affect the dung beetles' physiological condition in other tropical ecosystems.

In South America, Cerrado is a highly threatened savanna ecosystem due to the conversion of its natural landscapes into livestock (Garcia & Ballester, 2016). Thus, livestock farming has expanded widely in the Cerrado, where the increase in exotic pastures is responsible for 67% of the total land use change of the Cerrado biome (IBGE, 2018). In the Cerrado regions, dung beetle assemblages have been highly studied in natural and modified landscapes, such as introduced pastures (i.e., African grass – *Urochloa* spp.; Correa et al., 2020; Macedo et al., 2020; Maciel et al., 2023). Although we are aware of the biological consequences of Cerrado conversion into pasture for the dung beetle assemblage scale (Correa et al., 2020; Macedo et al., 2020; Maciel et al., 2023), there are no cues regarding the effects of this habitat transformation on individuals' physiological condition.

The aim of our study was to assess the effect of habitat type (native Cerrado vegetation and introduced pasture) on the physiological condition and body traits of males and females of *Phanaeus palaeno* Blanchard, 1845, a dung beetle that mainly occurs in Brazil, Bolivia, and Paraguay (Lizardo et al., 2022), but is particularly abundant in the Brazilian Cerrado (see Correa et al., 2016, 2020; Puker et al., 2014). Physiological condition was assessed through the estimation of individuals' dry body mass, fat mass, and muscle mass. Body traits were estimated through individual body size and males' horn length. Adult body size and males' horn length are affected by the quantity and quality of food resource available during individual larval stage (Moczek, 1998, 2002). Moreover, males' horn length is an important secondary sexual trait, being determinant for intrasexual contests to successfully obtain food and mate (Scholtz et al., 2009). Since *P. palaeno* presents a marked decrease on its abundance in non‐native habitats compared to native ones (Puker et al., 2014), we believe that such limitation in habitat distribution could be related to individual physiological conditions. If non‐native habitats constrain *P. palaeno* activity, we expect that beetles found in pastures have a lower physiological condition than to those found in the Cerrado biome. Moreover, the effects of anthropogenic habitats on physiological condition of dung beetles might be sex dependent (Salomão et al., 2020), while *Phanaeus* MacLeay, 1819 beetles have different nesting behaviors that depend on individual sex (Price & May, 2009). Thus, we expect that females and males have distinct physiological response to habitat type.

## 2 MATERIALS AND METHODS

### 2.1 Study site

The study was carried out in a region of Cerrado, in the Brazilian municipality of Aquidauana. The climate of the region, according to the Köppen classification, is Aw (tropical hot‐wet, with a rainy summer, and a dry winter; Alvares et al., 2014). Annual precipitation ranges from 1200 to 1300 mm and average annual temperature is 26°C. The studied area has been historically modified by livestock farming activities, the area being composed mainly of remnants of the native vegetation (Cerrado sensu stricto) and exotic pastures (*Urochloa* spp.) (Correa et al., 2019).

**FIGURE 1:**
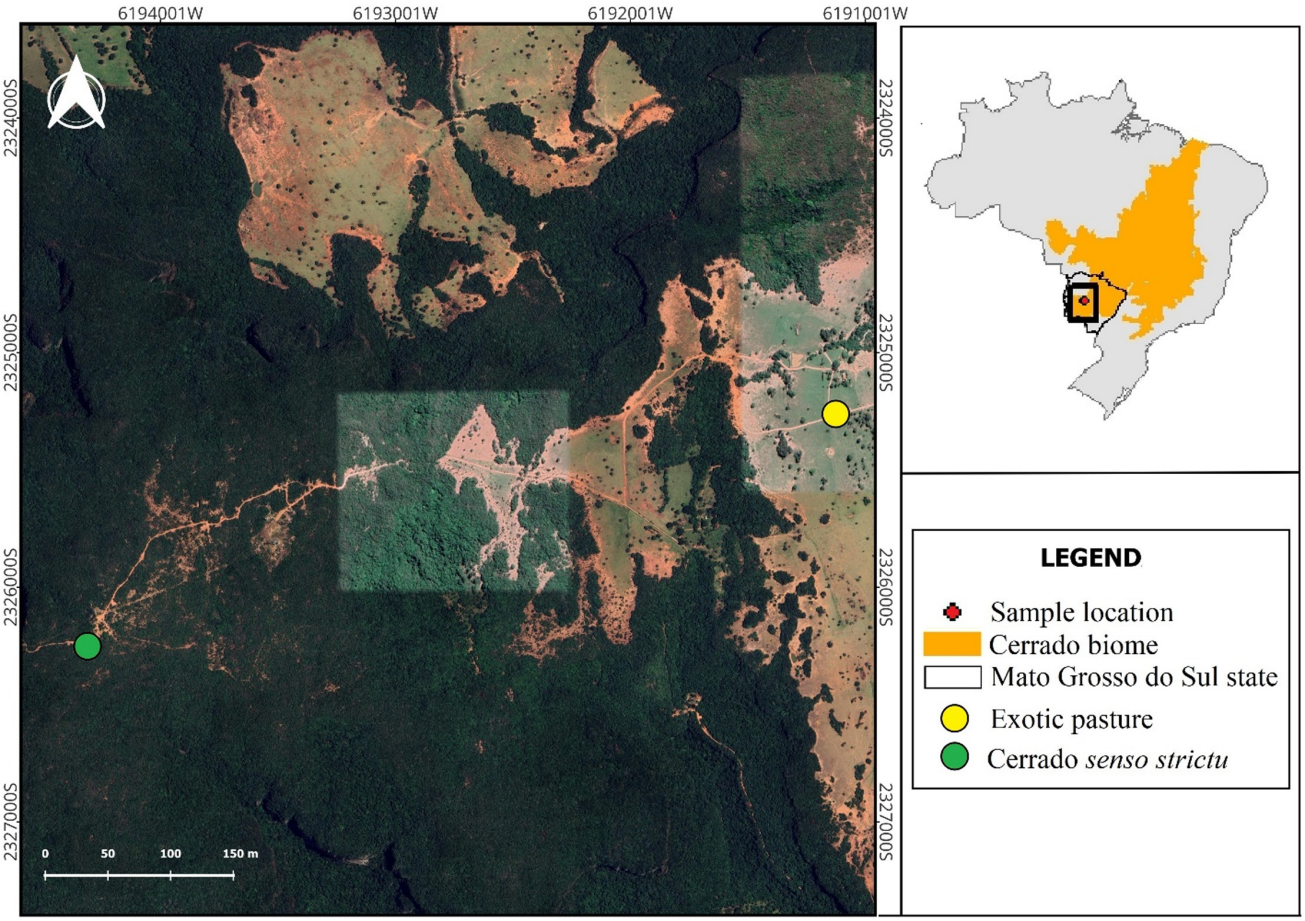
Localization of the studied area in Brazilian Cerrado (Aquidauana, Mato Grosso do Sul, Brazil), highlighting the sampled areas of exotic pasture and Cerrado sensu stricto.

Dung beetles were captured in two different habitats, in a pasture with exotic grass (*Urochloa* spp.) and a patch of Cerrado (Brazilian savanna). These two habitats were separated by 3 km (Figure 1).

1. Exotic pasture (20 ha; 20°26′25″ S, 55°36′57″ W, 470 m a.s.l.) is cultivated with African grasses (*Urochloa* ssp.), used for cattle production, with often use of veterinary drugs (e.g., ivermectin, usually 0.2 mg/kg an annual dose), with stocking rates between 0.8 and 1.3 livestock per hectare. They are managed in a rotational system where the cattle remain for ~20 days before being moved to other pastures and return after ~30 days.
2. Cerrado sensu stricto (30 ha; 20°26′55″ S, 55°38′40″ W, 480 m a.s.l.) is a conserved area with little sign of anthropogenic transformation. This site is characterized by a savanna woodland with a discontinuous canopy layer composed of trees and large shrubs (often 3–8 m; canopy covering 50%–90%) and a ground layer composed of grasses, herbs, and small shrubs (Fina & Monteiro, 2013). Typical wild animals of the Cerrado inhabit this patch, including anteaters, deers, manned wolfs, peccaries, and capybaras.

### 2.2 Dung beetle sampling

We collected the beetles every 2 weeks from October 2022 to January 2023 (during the rainy season), period of the highest activity of *P. palaeno* in this region (see Puker et al., 2014). In each habitat type, we installed five modified pitfall traps randomly distributed in the field, 50 m apart. Traps were built using a 3‐l plastic bottle with the top portion cut and placed upside down, so the bottle became funnel‐shaped (see Reis et al., 2023), to capture live beetles. These traps were buried in the soil without conserving liquid and remained installed in the field for a period of 24 h. Each trap was covered with a plastic lid to reduce desiccation of the bait (30 g of human feces) and avoid the accumulation of rainwater. The bait was placed in open plastic containers (50 mL) at the center of each trap using a wire as a bait holder.

Collections were repeated at the same exact location of the traps until to obtain enough individuals to perform physiological experiments. The individuals were separated by sex according to the presence of horns, where the females have no or only small horns, and the males have large horns (see Arnaud, 2002; Price & May, 2009; Puker et al., 2014). To avoid a possible mix (e.g., females and minor males), all females selected were individuals that did not present horns (see Table S1). Vouchers are deposited in the Laboratorio de Bioecologia de Scarabaeoidea (Scaralab) at UEMS (Universidade Estadual de Mato Grosso do Sul, Aquidauana, Brazil).

### 2.3 Dung beetles' physiological condition and body traits

Individuals were individually stored in plastic vials, being posteriorly sacrificed in freezers at −5°C, in which they were stored during 20 days before the estimation of dung beetles' physiological condition. We measured dry body mass, fat, and muscle mass as indicators of physiological condition. To obtain the dry body mass, the individuals were placed in a desiccator for 72 h and weighted to the nearest 0.0001 mg with a precision balance (Shimadzu). To extract the fat mass, we used the method of Marden (1989). Beetles were placed individually in falcon tubes containing 5 mL of chloroform for 48 h, then the individuals were placed in the desiccator again for 72 h and the difference between the body dry mass and this new mass was considered fat mass. Finally, the muscle mass of the beetles was extracted using the method of Plaistow and Siva‐Jothy (1996), in which individuals were placed in falcon tubes containing 0.2 M potassium hydroxide for 48 h, then the individuals were rinsed with water, re‐dried, and re‐weighed. The difference between the mass without fat and this new mass was considered muscle mass. Dung beetles of the current study were not emptied before being weighted, according to the observations carried out by Salomão et al. (2018). To control the effect of body size on physiological condition, we used relative values, which we obtained by dividing individual physiological variables by their body size.

Body size was estimated measuring from the clypeus to the pygidium, with a digital caliper with 0.01 mm of precision (see Puker et al., 2014). For this, the beetles were homogeneously set to be measured (i.e., all in a horizontal/similar position). Horn length of males was estimated measuring from the base to the apex of their horns, and the relative horn length was obtained by the division “horn length/body size.” Horn length was obtained from digital pictures taken using a digital camera (Canon SX240) and analyzed in Life Measurement software (Leica, Wetzlar, Germany). We measured physiological condition and body traits in 30 individuals per sex (female and male) in each habitat type (totaling 120 studied individuals). Although there is not an ideal number proposed for studies of body physiological conditions in dung beetles, previous studies in tropical ecosystems used 30–40 individuals per treatment (e.g., França, Barlow, et al., 2016; França, Louzada, et al., 2016; Salomão et al., 2020). A sampling effort of *n* = 60 is the minimum threshold recommended for ensuring an accurate estimation of dung beetle mean trait values (Griffiths et al., 2016). In this sense, we believe that the sampling effort that we used herein (*n* = 60 per habitat) could be appropriate to disentangle trustworthy ecological pattern and statistical analysis for this research.

### 2.4 Data analyses

The effect of habitat type on *P. palaeno* physiological conditions and body traits were assessed through Linear Models (LMs) and Weighted Least Square regressions. Sex was used as a covariable in the models. Habitat type, sex, and the interaction of habitat type × sex were the predictor variables. Body size, males' relative horn length, relative dry body mass, relative fat, and muscle mass were used as the response variables. For males' relative horn length, only habitat type was considered as predictor variable. Data were first checked for normality using the Shapiro–Wilk test (Shapiro & Wilk, 1965), dispersion of the residuals (Residual deviance/Residual df >2), outliers (visualized through Cooks' Distance), and homoscedasticity (tested with Fligner‐Killeen analysis). Individual body size, relative dry body mass, and fat mass showed data heteroscedasticity. For relative fat mass, heteroscedasticity was controlled by square root transformation of the data. For body size and relative dry body mass, data transformation did not solve the heteroscedasticity issue and thus, Weighted Least Square regressions were used. Models were done in R software version 4.2.0 (R Core Team, 2023).

## 3 RESULTS

From the total individuals analyzed, *P. palaeno* had a mean body size of 15.28 ± 1.53 mm (15.35 ± 1.05 mm among males and 15.21 ± 1.89 mm among females), with mean males' horn length of 7.42 ± 1.56 mm. Regarding physiological condition, individuals had a dry body mass of 18.74 ± 4.11 mg × 10^2^ (males: 19.43 ± 4.46 mg × 10^2^, females: 18.05 ± 6.89 mg × 10^2^), fat mass of 3.58 ± 2.26 mg × 10^2^ (males: 3.34 ± 2.09 mg × 10^2^, females: 3.83 ± 2.42 mg × 10^2^), and muscle mass of 3.62 ± 1.84 mg × 10^2^ (males: 3.87 ± 2.21 mg × 10^2^, females: 3.36 ± 1.36 mg × 10^2^). Cerrado encompassed beetles with a mean of 15.28 ± 1.58 mm of *P. palaeno* body size and males had a mean of 7.17 ± 1.17 of horn length, while pasture beetles had a mean of 15.27 ± 1.48 mm of body size and 7.67 ± 1.86 mm of males' horn length. Mean dry body mass, fat mass, and muscle mass of *P. palaeno* individuals in Cerrado were respectively 21.09 ± 5.22 mg × 10^2^, 4.56 ± 2.08 mg × 10^2^, and 3.02 ± 1.55 mg × 10^2^. In pasture, mean dry, fat, and muscle mass were 16.39 ± 5.46 mg × 10^2^, 2.60 ± 2.01 mg × 10^2^, and 4.21 ± 1.93 mg × 10^2^, respectively. For more detailed information, see Table S1.

**FIGURE 2:**
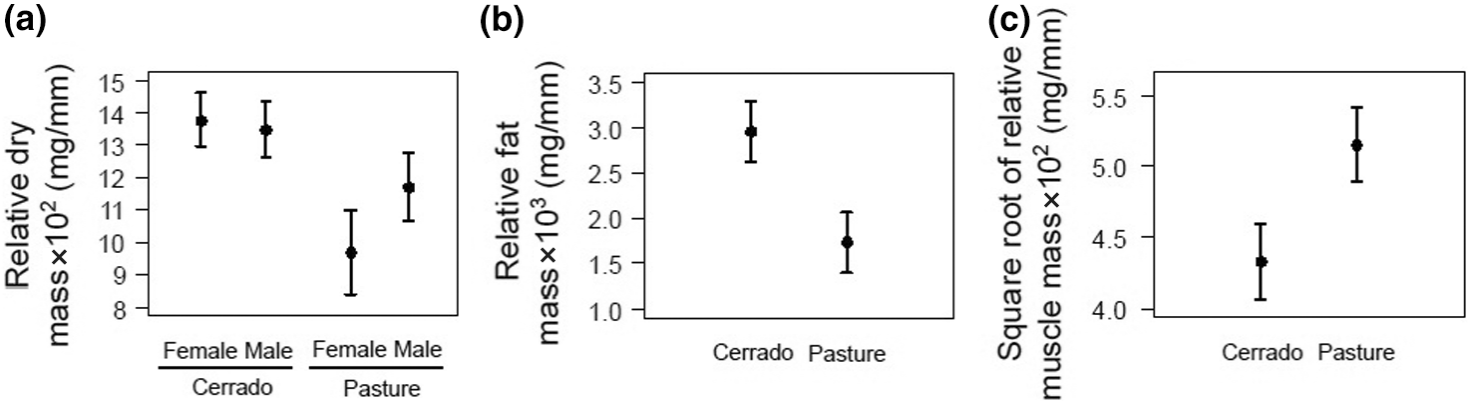
Statistical models presenting mean ± 95% confidence intervals of *Phanaeus palaeno* individuals' dry body mass of males and females sampled in Cerrado and pastures (a), and fat mass (b) and muscle mass (c) of individuals sampled in Cerrado and pastures.

Although habitat type did not affect *P. palaeno* individuals' body traits, physiological condition of these beetles differed between Cerrado and pasture (Table 1). Relative dry body mass was the only variable that was affected by the interaction of habitat type and sex, with Cerrado individuals presenting higher dry mass than pasture ones (Figure 2a). Moreover, although both male and female individuals had similar dry mass in Cerrado, males tended to be heavier than females in pastures (Figure 2a). Relative fat mass and muscle mass presented opposite patterns. While Cerrado comprised individuals with higher fat mass than those recorded in pasture (Figure 2b), muscle mass was lower in individuals collected in Cerrado than in pasture (Figure 2c).

**TABLE 1:** Models presenting the effect of habitat type (Cerrado and pasture), sex, and the interaction of habitat type and sex on *Phanaeus palaeno* body traits (body size and males' relative horn length) and physiological condition (relative dry body mass, fat mass, and muscle mass).

	Habitat type	Sex	Habitat type × sex
Body size (mm)	*F*_1,116_ = 0.11; *p* = .73	*F*_1,116_ = 0.23; *p* = .62	*F*_1,116_ = 2.65; *p* = .10
Males' relative horn size (mm)	*F*_1,58_ = 2.98; *p* = .08	NA	NA
Relative dry body mass (mg/mm)	***F***_**1,116**_ **= 29.18; *p* < .01**	*F*_1,116_ = 1.01; *p* = .31	***F***_**1,116**_ **= 4.93; *p* = .02**
Relative fat mass (mg/mm)	***F***_**1,116**_ **= 27.33; *p* < .01**	*F*_1,116_ = 2.13; *p* = .14	*F*_1,116_ = 3.31; *p* = .07
Relative muscle mass (mg/mm)	***F***_**1,116**_ **= 20.32; *p* < .01**	*F*_1,116_ = 2.11; *p* = .14	*F*_1,116_ = 3.53; *p* = .06

*Note*: Statistically significant models are shown in bold.

Abbreviation: NA, variable not included.

## 4 DISCUSSION

This study evaluated the dung beetle physiological and morphological responses to land use change in a tropical savanna. Although we did not find differences between dung beetle morphological traits between Cerrado and pastures, physiological conditions were affected by land use change. The biological consequences of Cerrado conversion into pasture are observed at the assemblage scale of dung beetles (Correa et al., 2020; Macedo et al., 2020; Maciel et al., 2023). Although *P. palaeno* spatial distribution is not constrained by anthropogenic habitats in Cerrado (Correa et al., 2020; Puker et al., 2014), pastures maintain individuals in poor physiological condition compared to those in conserved Cerrado habitats. Since individual health deterioration can precedes population declines in disturbed environments (Salomão et al., 2020), the lower physiological condition found in the individuals of the pasturelands could affect its individual survival and fitness (Chastel et al., 1995), which consequently may impact the population dynamics of *P. palaeno* in pasturelands in a longer term. Indeed, *P. palaeno* is considered of low importance to Brazilian pastures due to its low abundance in this ecosystem (Tissiani et al., 2017). Thus, despite the presence of *P. paleano* beetles in the studied pasture as reported 10 years before our collections (see Puker et al., 2014), the poor physiological condition of the individuals can anticipate future decreases in its populations (Fefferman & Romero, 2013; Wikelski & Cooke, 2006). Therefore, it is crucial to conduct ongoing temporal studies in this tropical pastureland, to better understand how *P. palaeno* body conditions affect its ability to successfully adapt to pastures in the long term.

Since pastures represent a landscape that challenges biodiversity maintenance in the tropics (Leão‐Gomes & Vasconcelos, 2023; Santos‐Gally & Boege, 2022), we expected a higher value of physiological condition in conserved vegetation (Cerrado). We found contrasting and interesting results, in which Cerrado presented *P. palaeno* individuals with higher dry and fat mass but lower muscle mass compared to the individuals recorded in pastures. Vegetation structure, food availability, and climatic conditions are some of the most determining factors that modulate animals' body mass (Aubry et al., 2013; Baines et al., 2015; Battles et al., 2013; McKinnon et al., 2015). Each animal species has different strategies regarding energetic storage, which results in different physiological responses towards environmental conditions. For example, the forest‐dwelling *Onthophagus rhinolophus* Harold, 1869 present individuals with higher muscle mass in landscapes with more edge amounts, while the pasture‐dweller *Onthophagus batesi* Howden & Cartwright, 1963 present an opposite trend (Salomão et al., 2018). Moreover, there are mammals and birds that are not affected, or even increase their physiological conditions in anthropized habitat compared to conserved ones (e.g., Aubry et al., 2013; Boulanger et al., 2013; McKinnon et al., 2015). Considering the natural history and foraging strategies of dung beetle species, the contrasting effects observed herein lead us to some important statements. As fat mass is the fuel of flight of insect species (Baines et al., 2015; Gade et al., 2004), the higher fat mass observed in Cerrado compared to pastures could suggest that *P. palaeno* individuals from these sites has more energetic stores that allow them to flight longer distances than the populations recorded in pastures. Among *Phanaeus* beetles, flight is often related to find food resources and consequently to mate (Price & May, 2009). Since *P. palaeno* feeds on cattle dung (Tissiani et al., 2017), we believe that the high spatial availability of dung resource in pasture could reduce the need of these beetles to flight long distances to obtain food resource when compared to Cerrado. Thus, considering the potentially more random food availability in Cerrado (i.e., dung from native mammals) when compared to pastures, one hypothesis is that individuals inhabiting Cerrado would need higher fat reserves to allow them to forage during longer distances.

Regarding the higher muscle mass of *P. palaeno* in pastures compared to Cerrado, two plausible hypotheses should be tested to clarify this pattern. Food quality drives dung beetle physiological condition (Servín‐Pastor et al., 2021), and dung beetles that are using pastures potentially are using cattle dung, which is markedly distinct from dung types from Cerrado mammals (e.g., *Chrysocyon brachyurus*, *Nasua nasua*, *Puma concolor*; Pônzio et al., 2022). Thus, food quality could be increasing muscle mass of pasture *P. palaeno* compared to Cerrado ones. A second hypothesis is that environmental conditions may filter dung beetle intraspecific traits (Giménez‐Gómez et al., 2020; Salomão et al., 2021). Thus, the higher muscle mass in pastures could suggest that dung beetles in this habitat faces competitively more aggressive episodes when compared to those that inhabit Cerrado. Moreover, considering the more compacted and limiting conditions of soils in pasture, dung beetles with more muscle mass could perform their nests more efficiently compared to individuals with low muscle reserves (Halffter et al., 1992). The contrasting results observed herein provide a heterogeneous scenario, with individuals from each habitat presenting clearly distinct physiological condition. In a study performed in southern Mexico, individuals of *Canthon cyanellus* LeConte, 1859 apparently presented a population segregation according to physiological condition, age, and sex among habitat types (Salomão et al., 2021), suggesting that individuals could selected the best suited habitat according to their individual condition. To better understand how populations of dung beetles move across habitats and how their body masses are used and change throughout time, mark recapture and long‐term studies should be addressed.

We did not find significant differences in *P. palaeno* individuals' body size between Cerrado and pastures sites. Body size is determined during larval development, which does not change during adulthood (Karino et al., 2004; Moczek, 1998). In Neotropical rainforests of Mexico, landscape diversity, connectivity and the amount of edges affect body size of individuals of native species that inhabit both pastures and conserved habitats (Salomão et al., 2018). Moreover, dung beetles in more conserved habitats (primary forests located in mainland) were larger than those collected in disturbed sites (secondary forests located in islands) in an insular landscape of Amazonian region (Cerqueira et al., 2023). According to these studies and our data, we can suggest that individual body size may be driven by landscape shifts and habitats that strongly limits individual dispersion, as the abovementioned Amazonian islands. In the scenario of our study, we have a native arboreous habitat (Cerrado) and an open‐canopy one (pasture). Considering that *P. palaeno* inhabit both conserved and disturbed sites in Cerrado (Correa et al., 2020; Puker et al., 2014), we could consider that its populations are able to move across these two habitats – as observed in other tropical dung beetles (Barretto et al., 2023; Cultid‐Medina et al., 2015). Under this assumption, the similar body sizes of individuals found in Cerrado and pasture could indicate that intraspecific competitive pressures that depend on beetle body size (e.g., competition for partners) are similar between those habitats. Nonetheless, under the scenario that the populations of *P. palaeno* tend to not move across different habitats, we could suggest that resource availability for larval development is similar between the Cerrado and pastures. To have stronger cues regarding the distribution of *P. palaeno* individuals regarding their body size, it is important to understand their dispersal patterns throughout Cerrado landscapes.

In scarabaeid beetles, larger‐horned males have a higher probability to win male–male contests and to successfully breed (Scholtz et al., 2009; Sol et al., 2021). Our data demonstrate that males' horn length was similar between Cerrado and pasture populations. From a biological conservation perspective, this result suggests that males' main sexual trait of the focal species is not restrained due to habitat transformation. Such results, together with the body size and physiology trend found herein, allow us to propose an interesting statement: while population body traits can blur any population response towards habitat transformation, physiological traits were clearly affected by environmental quality. Since the effects of environmental quality on dung beetles' diversity and individual condition is mostly assessed considering species body size (e.g., Correa et al., 2019; Souza et al., 2020), our results suggest that physiology could present finer comprehension of habitat transformation trends on individual condition. Although the estimation of physiological condition is more complex than species body size, we suggest that future studies that aim to present complex and finer responses of dung beetles should include physiological data.

## 5 CONCLUSIONS

Until now, there has been very little information on the effects of environmental disturbances on dung beetle body physiological conditions in Neotropical regions (see França, Barlow, et al., 2016; França, Louzada, et al., 2016; Salomão et al., 2018, 2020). Our results show that individual physical condition is sensitive to conversion of native savannah into pasturelands, highlighting the importance of body physical condition measurements to predict how species respond to environmental change (see Cooke et al., 2013; França, Barlow, et al., 2016; França, Louzada, et al., 2016; Salomão et al., 2018, 2020). As we found contrasting results to physiological characteristics evaluated (e.g., dry body mass, fat mass, and muscle mass), this result emphasizes the importance of using more than one physiological metric to evaluate the effect of land uses on dung beetle body physical condition. Finally, understanding how the land use change effects on the species' body condition is essential to maintain not only abundant species, but also healthy populations of dung beetles in human‐modified landscapes (Salomão et al., 2018).

## AUTHOR CONTRIBUTIONS

**César M. A. Correa:** Conceptualization (equal); data curation (equal); formal analysis (equal); investigation (equal); methodology (equal); project administration (equal); supervision (equal); validation (equal); writing – original draft (equal); writing – review and editing (equal). **Kalel Caetano da Silva:** Data curation (equal); investigation (equal); methodology (equal). **Pedro Lucas Moreira de Oliveira:** Data curation (equal); investigation (equal); methodology (equal). **Renato Portela Salomão:** Data curation (equal); formal analysis (equal); writing – original draft (equal); writing – review and editing (equal).

## CONFLICT OF INTEREST STATEMENT

The authors declare no conflicts of interest.

## Supporting information


Table S1

## Data Availability

The data that support the findings of this study are available in the supplementary material of this article.
