# Supplementary material for: The conversion of native savannah into pasturelands does not affect exclusively species diversity: Effects on physiological condition of a highly abundant dung beetle species
Source: Ecol Evol. 2023 Nov 20;13(11):e10752. doi: 10.1002/ece3.10752 (PMC10659944; doi:10.1002/ece3.10752)
Supplement: Supplementary file 1 — Table S1 [file ECE3-13-e10752-s001.docx]

**Table S1.** Morphological and physiological traits of individuals of *Phanaeus palaeno* (Coleoptera: Scarabaeinae) collected in Aquidauana, Mato Grosso do Sul, Brazil.

| **Individual** | **Habitat** | **Sex** | **Horn length (mm)** | **Body length (mm)** | **Body Dry Mass (mg)** | **Fat Mass**  **(mg)** | **Muscle Mass (mg)** |
| --- | --- | --- | --- | --- | --- | --- | --- |
| P1 | Pasture | Male | 8.69 | 16.42 | 0.1992 | 0.0241 | 0.0814 |
| P2 | Pasture | Male | 8.19 | 15.86 | 0.1688 | 0.046 | 0.055 |
| P3 | Pasture | Male | 7.86 | 15.17 | 0.1837 | 0.0422 | 0.0388 |
| P4 | Pasture | Male | 9.37 | 16.06 | 0.1823 | 0.0094 | 0.0407 |
| P5 | Pasture | Male | 9.33 | 15 | 0.1098 | 0.0029 | 0.0343 |
| P6 | Pasture | Male | 8.01 | 15.45 | 0.1226 | 0.0036 | 0.0506 |
| P7 | Pasture | Male | 7.62 | 13.83 | 0.1323 | 0.0120 | 0.0431 |
| P8 | Pasture | Male | 7.72 | 12.92 | 0.133 | 0.0278 | 0.0457 |
| P9 | Pasture | Male | 7.77 | 16.01 | 0.2016 | 0.0295 | 0.0384 |
| P10 | Pasture | Male | 2.69 | 13.75 | 0.1397 | 0.0374 | 0.0420 |
| P11 | Pasture | Male | 8.93 | 15.12 | 0.2039 | 0.0249 | 0.0642 |
| P12 | Pasture | Male | 7.82 | 13.97 | 0.1863 | 0.0833 | 0.0160 |
| P13 | Pasture | Male | 9.82 | 14.21 | 0.0856 | 0.0035 | 0.0303 |
| P14 | Pasture | Male | 7.82 | 16.57 | 0.2329 | 0.0268 | 0.1524 |
| P15 | Pasture | Male | 8.79 | 14.94 | 0.1529 | 0.0043 | 0.0517 |
| P16 | Pasture | Male | 9.43 | 15.7 | 0.2231 | 0.0470 | 0.0458 |
| P17 | Pasture | Male | 7.44 | 17.61 | 0.2146 | 0.0161 | 0.0586 |
| P18 | Pasture | Male | 8.15 | 14.89 | 0.189 | 0.0263 | 0.0457 |
| P19 | Pasture | Male | 9.49 | 14.99 | 0.2279 | 0.0414 | 0.0507 |
| P20 | Pasture | Male | 7.25 | 17.05 | 0.2293 | 0.0251 | 0.0667 |
| P21 | Pasture | Male | 6.85 | 14.31 | 0.2235 | 0.0562 | 0.0501 |
| P22 | Pasture | Male | 7.65 | 14.51 | 0.1145 | 0.0052 | 0.0357 |
| P23 | Pasture | Male | 5.55 | 15.79 | 0.1412 | 0.0163 | 0.0344 |
| P24 | Pasture | Male | 1.72 | 14.38 | 0.1178 | 0.0056 | 0.0367 |
| P25 | Pasture | Male | 4.54 | 13.03 | 0.1332 | 0.0146 | 0.0463 |
| P26 | Pasture | Male | 7.63 | 16.23 | 0.2345 | 0.0382 | 0.0302 |
| P27 | Pasture | Male | 9.1 | 15.97 | 0.2637 | 0.0481 | 0.0229 |
| P28 | Pasture | Male | 9.16 | 13.32 | 0.191 | 0.0247 | 0.0333 |
| P29 | Pasture | Male | 8.28 | 15.11 | 0.2097 | 0.0365 | 0.0314 |
| P30 | Pasture | Male | 7.65 | 15.44 | 0.1857 | 0.0198 | 0.0442 |
| P31 | Pasture | Female | NA | 12.16 | 0.2398 | 0.0808 | 0.0488 |
| P32 | Pasture | Female | NA | 12.75 | 0.064 | 0.0177 | 0.0114 |
| P33 | Pasture | Female | NA | 17.58 | 0.2057 | 0.0443 | 0.0382 |
| P34 | Pasture | Female | NA | 14.03 | 0.1077 | 0.0332 | 0.0252 |
| P35 | Pasture | Female | NA | 13.12 | 0.0691 | 0.0076 | 0.0274 |
| P36 | Pasture | Female | NA | 16.52 | 0.1632 | 0.0221 | 0.0444 |
| P37 | Pasture | Female | NA | 18.45 | 0.1169 | 0.0084 | 0.0479 |
| P38 | Pasture | Female | NA | 13.62 | 0.1037 | 0.0149 | 0.0375 |
| P39 | Pasture | Female | NA | 13.83 | 0.125 | 0.0203 | 0.0303 |
| P40 | Pasture | Female | NA | 15.77 | 0.1454 | 0.0226 | 0.0382 |
| P41 | Pasture | Female | NA | 13.14 | 0.1799 | 0.0391 | 0.0602 |
| P42 | Pasture | Female | NA | 16.53 | 0.1924 | 0.0346 | 0.0384 |
| P43 | Pasture | Female | NA | 15.75 | 0.1902 | 0.0143 | 0.0409 |
| P44 | Pasture | Female | NA | 15.14 | 0.065 | 0.0075 | 0.0242 |
| P45 | Pasture | Female | NA | 15.38 | 0.0617 | 0.0043 | 0.0211 |
| P46 | Pasture | Female | NA | 15.33 | 0.0693 | 0.0067 | 0.0338 |
| P47 | Pasture | Female | NA | 13.77 | 0.0822 | 0.0036 | 0.0297 |
| P48 | Pasture | Female | NA | 15.21 | 0.2085 | 0.0543 | 0.0416 |
| P49 | Pasture | Female | NA | 16.24 | 0.1498 | 0.0122 | 0.0434 |
| P50 | Pasture | Female | NA | 16.22 | 0.1512 | 0.0175 | 0.0363 |
| P51 | Pasture | Female | NA | 15.69 | 0.1543 | 0.0091 | 0.0396 |
| P52 | Pasture | Female | NA | 16.61 | 0.2249 | 0.0364 | 0.0459 |
| P53 | Pasture | Female | NA | 17.12 | 0.2362 | 0.0893 | 0.0292 |
| P54 | Pasture | Female | NA | 17.18 | 0.1282 | 0.0042 | 0.0293 |
| P55 | Pasture | Female | NA | 17.95 | 0.2462 | 0.0279 | 0.0555 |
| P56 | Pasture | Female | NA | 16.07 | 0.2612 | 0.0598 | 0.0593 |
| P57 | Pasture | Female | NA | 17.03 | 0.1306 | 0.0161 | 0.0237 |
| P58 | Pasture | Female | NA | 16.94 | 0.1629 | 0.0244 | 0.0409 |
| P59 | Pasture | Female | NA | 16.18 | 0.1732 | 0.0091 | 0.0498 |
| P60 | Pasture | Female | NA | 11.78 | 0.0958 | 0.0240 | 0.0215 |
| C1 | Cerrado | Male | 7.23 | 14.44 | 0.2084 | 0.0339 | 0.0739 |
| C2 | Cerrado | Male | 6.94 | 14.29 | 0.1738 | 0.0185 | 0.0409 |
| C3 | Cerrado | Male | 8.31 | 16.71 | 0.2156 | 0.0422 | 0.0164 |
| C4 | Cerrado | Male | 6.59 | 14.19 | 0.1582 | 0.0169 | 0.0253 |
| C5 | Cerrado | Male | 6.61 | 14.76 | 0.1737 | 0.0334 | 0.0146 |
| C6 | Cerrado | Male | 8.08 | 15.36 | 0.2163 | 0.0616 | 0.0175 |
| C7 | Cerrado | Male | 7.81 | 15.35 | 0.1829 | 0.0369 | 0.0202 |
| C8 | Cerrado | Male | 8.07 | 15.61 | 0.2054 | 0.0224 | 0.0310 |
| C9 | Cerrado | Male | 7.72 | 15.72 | 0.2341 | 0.0585 | 0.0263 |
| C10 | Cerrado | Male | 9.07 | 16.21 | 0.2233 | 0.0264 | 0.0252 |
| C11 | Cerrado | Male | 4.24 | 15.17 | 0.2087 | 0.0413 | 0.0125 |
| C12 | Cerrado | Male | 7.32 | 14.74 | 0.1626 | 0.0159 | 0.0216 |
| C13 | Cerrado | Male | 6.54 | 14.86 | 0.1751 | 0.0350 | 0.0207 |
| C14 | Cerrado | Male | 8.79 | 15.22 | 0.1916 | 0.0344 | 0.0234 |
| C15 | Cerrado | Male | 8.43 | 15.87 | 0.2488 | 0.0668 | 0.0220 |
| C16 | Cerrado | Male | 5.46 | 16.39 | 0.2414 | 0.0393 | 0.0734 |
| C17 | Cerrado | Male | 5.42 | 16.69 | 0.2204 | 0.0416 | 0.0248 |
| C18 | Cerrado | Male | 7.54 | 15.27 | 0.2194 | 0.0510 | 0.0686 |
| C19 | Cerrado | Male | 7.69 | 14.00 | 0.2006 | 0.0433 | 0.0286 |
| C20 | Cerrado | Male | 4.51 | 16.21 | 0.2259 | 0.0633 | 0.0273 |
| C21 | Cerrado | Male | 6.54 | 14.66 | 0.2178 | 0.0726 | 0.0177 |
| C22 | Cerrado | Male | 6.54 | 14.65 | 0.1748 | 0.0191 | 0.0214 |
| C23 | Cerrado | Male | 7.06 | 16.55 | 0.2394 | 0.0223 | 0.0249 |
| C24 | Cerrado | Male | 7.35 | 16.66 | 0.1859 | 0.0198 | 0.0241 |
| C25 | Cerrado | Male | 7.12 | 16.74 | 0.3330 | 0.1039 | 0.0223 |
| C26 | Cerrado | Male | 8.05 | 14.93 | 0.1969 | 0.0435 | 0.0192 |
| C27 | Cerrado | Male | 8.04 | 15.97 | 0.1833 | 0.0146 | 0.039 |
| C28 | Cerrado | Male | 6.07 | 16.3 | 0.2016 | 0.0312 | 0.0557 |
| C29 | Cerrado | Male | 7.43 | 17.17 | 0.2966 | 0.075 | 0.039 |
| C30 | Cerrado | Male | 8.56 | 16.88 | 0.2143 | 0.0246 | 0.032 |
| C31 | Cerrado | Female | NA | 14.23 | 0.1453 | 0.0326 | 0.0479 |
| C32 | Cerrado | Female | NA | 13.33 | 0.1670 | 0.0379 | 0.0608 |
| C33 | Cerrado | Female | NA | 11.3 | 0.1194 | 0.0322 | 0.0185 |
| C34 | Cerrado | Female | NA | 13.94 | 0.1939 | 0.0316 | 0.0394 |
| C35 | Cerrado | Female | NA | 14.62 | 0.1872 | 0.0505 | 0.0227 |
| C36 | Cerrado | Female | NA | 13.34 | 0.1925 | 0.0508 | 0.0176 |
| C37 | Cerrado | Female | NA | 13.36 | 0.1377 | 0.0343 | 0.0221 |
| C38 | Cerrado | Female | NA | 12.29 | 0.1110 | 0.0256 | 0.0176 |
| C39 | Cerrado | Female | NA | 17.2 | 0.3002 | 0.0698 | 0.0282 |
| C40 | Cerrado | Female | NA | 16.75 | 0.2414 | 0.0622 | 0.0627 |
| C41 | Cerrado | Female | NA | 14.86 | 0.1613 | 0.0342 | 0.0295 |
| C42 | Cerrado | Female | NA | 14.21 | 0.1615 | 0.0325 | 0.0221 |
| C43 | Cerrado | Female | NA | 13.94 | 0.2121 | 0.0734 | 0.0175 |
| C44 | Cerrado | Female | NA | 12.37 | 0.1631 | 0.0425 | 0.0244 |
| C45 | Cerrado | Female | NA | 16.49 | 0.2671 | 0.0736 | 0.0175 |
| C46 | Cerrado | Female | NA | 16.75 | 0.2723 | 0.0562 | 0.0162 |
| C47 | Cerrado | Female | NA | 16.87 | 0.2915 | 0.0742 | 0.0251 |
| C48 | Cerrado | Female | NA | 13.83 | 0.1605 | 0.0427 | 0.0188 |
| C49 | Cerrado | Female | NA | 12.67 | 0.1233 | 0.0315 | 0.0328 |
| C50 | Cerrado | Female | NA | 11.31 | 0.1155 | 0.0239 | 0.0087 |
| C51 | Cerrado | Female | NA | 16.84 | 0.2538 | 0.0586 | 0.0511 |
| C52 | Cerrado | Female | NA | 15.29 | 0.2234 | 0.0668 | 0.0191 |
| C53 | Cerrado | Female | NA | 16.39 | 0.2682 | 0.0606 | 0.0367 |
| C54 | Cerrado | Female | NA | 15.39 | 0.2575 | 0.0733 | 0.0537 |
| C55 | Cerrado | Female | NA | 17.00 | 0.2988 | 0.0929 | 0.0075 |
| C56 | Cerrado | Female | NA | 18.59 | 0.3153 | 0.0831 | 0.0325 |
| C57 | Cerrado | Female | NA | 15.2 | 0.1734 | 0.0253 | 0.0375 |
| C58 | Cerrado | Female | NA | 15.68 | 0.2266 | 0.0375 | 0.0364 |
| C59 | Cerrado | Female | NA | 19.07 | 0.2986 | 0.0529 | 0.0458 |
| C60 | Cerrado | Female | NA | 16.64 | 0.2873 | 0.0705 | 0.0365 |
